# Supplementary material for: Metabolome and Transcriptome Analyses Reveal Flower Color Differentiation Mechanisms in Various Sophora japonica L. Petal Types
Source: Biology (Basel). 2023 Nov 25;12(12):1466. doi: 10.3390/biology12121466 (PMC10740404; doi:10.3390/biology12121466)
Supplement: Supplementary file 1 [file biology-12-01466-s001.zip › Description of supplementary materials.pdf]

The following is the supplementary data related to this article:

Supplementary Figure S1. Correlation analysis among the various petal types color difference values (L*, a*, and b*) in *S. japonica* 'AM' mutant at different flower developmental stages.

Supplementary Figure S2. Heat map of correlation between anthocyanin content and expression of anthocyanin synthesis structural genes.

Supplementary Figure S3. Phylogenetic tree of *SjbHLH1* amino acid sequences and otherpublished anthocyanin-related bHLHs. The GenBank accession numbers are:AhNAI1 (LOC112775145), AibHLH18-like (LOC107614694), AhbHLH18 (LOC112712823), AdbHLH18-like (LOC107463717),AsNAI1-like (LOC130976602), LjbHLH18-like (LOC130742633), GmbHLH25-like (LOC100795184), GmbHLH18 (LOC100809223), ApbHLH18-like (LOC113854852), CcbHLH25 (LOC109808911), PabHLH19-like ((LOC114740048), GmbHLH2 (LOC100809888), VubHLH18-like (LOC124835930), VabHLH18 (LOC108320439), VuNAI1 (LOC114166803), GsbHLH18-like (LOC114424526), GmNAI1 (LOC100809956), CcbHLH18 (LOC109818143), ApNAI1-like (LOC1138571390).

Supplementary Table S1. Anthocyanin candidate gene primers.

Supplementary Table S2. qRT-PCR reaction system and reaction protocol.

Supplementary Table S3. Color variation of different petal types in S. japonica 'AM' mutant at different flower developmental stages.

Supplementary Table S4. Reference genome sequence alignment.
