# Supplementary material for: Metabolome and Transcriptome Analyses Reveal Flower Color Differentiation Mechanisms in Various Sophora japonica L. Petal Types
Source: Biology (Basel). 2023 Nov 25;12(12):1466. doi: 10.3390/biology12121466 (PMC10740404; doi:10.3390/biology12121466)
Supplement: Supplementary file 1 [file biology-12-01466-s001.zip › Supplementary Table.pdf]

Table S1. Anthocyanin candidate gene primers.

Gene name	Gene ID	Forward primers	Reverse primers
SjUGT79B1	GHAGGene01140	5'GAAGCAGAAGCAGAAGCAGAA3'	5'CGTTGATGGGTGACTCAGTATC3'
SjDFR	GHAGGene00696	5'CAGCACTCTGTGAGGCAACT3'	5'CCAACACTGGCATTCAAGAACT3'
SjFLS	GHAGGene21877	5'GAGCAACCAGGCATCACAA3'	5'CTAGAATCTCACGCAGGACTT3'
SjF3H	GHAGGene06185	5'GAGGTGTTGTCAGAGGCAATG3'	5'CCAGTGTGAGGTCAGGTTGT3'
SjCHS1	GHAGGene19992	5'ATGGTGACCGTTGAGGAGAT3'	5'GTGCTCGCTGTCTGTGATG3'
SjCHS2	GHAGGene04222	5'TGGTGAGCGTGGAGGAGAT3'	5'CTGTTGGTAATGCGGAAGTAGT3'
SjF3'5'H1	GHAGGene03412	5'ATGGCGTCCGAAGGCATT3'	5'GCATTGTTCAAGGCGATGTCA3'
SjF3'5'H2	GHAGGene27644	5'AGCAGCAAGTGATGATACCTAC3'	5'AGCAGGACCAAGACGCATA3'
SjPAL	GHAGGene09401	5'CAGGCAAGCAACCAAGATGA3'	5'CCAGACGAGTGTTATCCATTGA3'
SjCHI	GHAGGene13892	5'GGAGCTGGAAGAGAAGGATGA3'	5'CCTCACGGCAGTCTCAATCT3'
SjANS	GHAGGene01871	5'CACAATATGGTGCCAGGTCTG3'	5'ACTTGCCGTTGCTCAGGAT3'
SjMYB	GHAGGene24051	5'TGTAGGCTAAGATGGCTGAACT3'	5'TTGGCAGTCCTTCCTGGTAG3'
SjbHLH1	GHAGGene13386	5'CACCATCCACGACGATTCCT3'	5'GCCGCTGAACTTGCTTCTG3'
SjbHLH2	GHAGGene00848	5'TGGCGTGAGTGATGGTGAT3'	5'TAATCTTGCTTAGGAGGAGGCT3'
SjM-type_MADS	GHAGGene06148	5'AACTCGGACTGATACCTGTAGG3'	5'CTGTTGATTCCTCGTCTCCTTC3'
SjIAA	GHAGGene03678	5'GGCAGTGATGAGACCGAGAA3'	5'GTTGCTCTTGCTTATGGACTCT3'
ubiquitin		5'CGAGGTGACCGAGCAGCAAAGAG3'	5'TTGCCACATCCACCACCGTCC3'

Table S2. qRT-PCR reaction system and reaction protocol.

The reaction system		The reaction procedure		
Reagents	Usage amount	Pre-denaturation	94 ℃ 30 s	1 个 Cycle
TB Green Fast qPCR Mix（2X）	10 μL	PCR reaction	94 ℃ 5 s	40 个 Cycles
PCR Forward Primer（10 μM）	0.8 μL		60 ℃ 10 s	
PCR Reverse Primer（10 μM）	0.8 μL		95 ℃ 5 s	
DNA template	2 μL	Dissolve	60 ℃ 1 min	1 个 Cycle
Rnase free dH_2O	6.4 μL		95 ℃	
Total	20 μL	Cooling	50 ℃ 30 s	1 个 Cycle

Table S3. Color variation of different petal types in *S. japonica* 'AM' mutant at different flower developmental stages.

Petal types	L*	a*	b*
S1 QB	90.76±4.46	-2.54±0.09	36.31±2.56
S1 YB	80.02±3.79	11.00±1.09	17.42±0.27
S1 LGB	83.99±3.28	6.41±0.28	26.77±2.38
S2 QB	86.93±4.64	-4.50±0.18	31.92±0.93
S2 YB	73.26±0.59	15.46±2.03	23.30±1.38
S2 LGB	75.91±0.67	7.15±0.05	29.04±0.12
S3 QB	78.74±2.52	-2.70±0.34	27.34±1.59
S3 YB	75.73±3.35	6.53±0.35	18.78±0.44
S3 LGB	78.62±0.52	4.40±0.06	22.14±1.29
S4 QB	93.37±3.53	-3.97±0.09	21.57±0.58
S4 YB	85.19±1.96	4.36±0.19	16.06±0.69
S4 LGB	86.05±0.76	3.59±0.14	19.86±0.68

Table S4. Reference genome sequence alignment.

Sample name	Clean reads(M)	Q20 rate(%)	Q30 rate(%)	Unique_mapped (%)	Multiple_mapped (%)	Unmapped (%)
S1QB1	6,670.83	97.38	92.26	93.21	2.85	3.94
S1QB2	6,671.18	96.98	91.25	93.82	2.88	3.30
S1QB3	6,672.28	96.81	90.82	93.77	2.85	3.38
S1YB1	6,445.19	96.79	90.84	94.42	2.74	2.84
S1YB2	6,673.22	97.06	91.45	94.34	2.72	2.94
S1YB3	6,666.92	96.65	90.44	94.26	2.56	3.18
S1LGB1	6,667.74	96.51	90.00	94.57	2.46	2.97
S1LGB2	6,669.05	96.50	89.90	95.01	2.40	2.59
S1LGB3	6,671.27	96.66	90.48	94.63	2.37	3.00
S2QB1	6,667.22	96.77	90.74	94.25	2.71	3.04
S2QB2	6,669.68	96.45	89.77	92.48	2.80	4.72
S2QB3	6,669.77	96.79	90.86	94.25	2.68	3.07
S2YB1	6,669.45	97.36	92.36	94.78	2.51	2.71

Sample name	Clean reads(M)	Q20 rate(%)	Q30 rate(%)	Unique_mapped (%)	Multiple_mapped (%)	Unmapped (%)
S2YB2	6,672.65	97.01	91.41	94.66	2.57	2.77
S2YB3	6,667.18	96.59	90.14	94.81	2.45	2.74
S2LGB1	6,670.67	96.94	91.26	94.79	2.49	2.72
S2LGB2	6,147.63	96.48	89.93	94.84	2.51	2.65
S2LGB3	6,672.16	96.70	90.43	94.92	2.49	2.59
S3QB1	6,670.31	96.03	88.85	92.13	3.10	4.77
S3QB2	6,672.28	96.45	89.92	93.47	3.06	3.47
S3QB3	6,667.19	96.98	91.32	86.88	3.05	10.07
S3YB1	6,667.03	96.48	89.83	93.45	3.06	3.49
S3YB2	6,669.77	97.13	91.76	93.83	3.02	3.15
S3YB3	6,670.61	96.98	91.33	93.29	2.97	3.74
S3LGB1	6,671.87	96.91	91.12	93.62	2.91	3.47
S3LGB2	6,042.14	96.38	89.67	93.99	2.89	3.12
S3LGB3	6,666.67	96.91	91.14	93.85	2.89	3.26
S4QB1	6,672.92	97.30	92.18	93.20	3.07	3.73
S4QB2	6,672.57	97.15	91.82	92.77	3.31	3.92
S4QB3	6,669.25	96.83	91.02	90.42	7.94	19.50
S4YB1	6,669.44	96.98	91.24	92.97	3.24	3.79
S4YB2	6,669.62	97.11	91.69	92.60	3.30	4.10
S4YB3	6,031.44	96.83	90.97	93.40	3.36	3.24
S4LGB1	6,033.82	96.71	90.54	93.74	3.08	3.18
S4LGB2	6,664.89	96.71	90.52	93.57	3.20	3.23
S4LGB3	6,669.21	96.86	90.88	93.78	2.95	3.27
