# Supplementary figures and images for: Metabolome and Transcriptome Analyses Reveal Flower Color Differentiation Mechanisms in Various Sophora japonica L. Petal Types
Source: Biology (Basel). 2023 Nov 25;12(12):1466. doi: 10.3390/biology12121466 (PMC10740404; doi:10.3390/biology12121466)

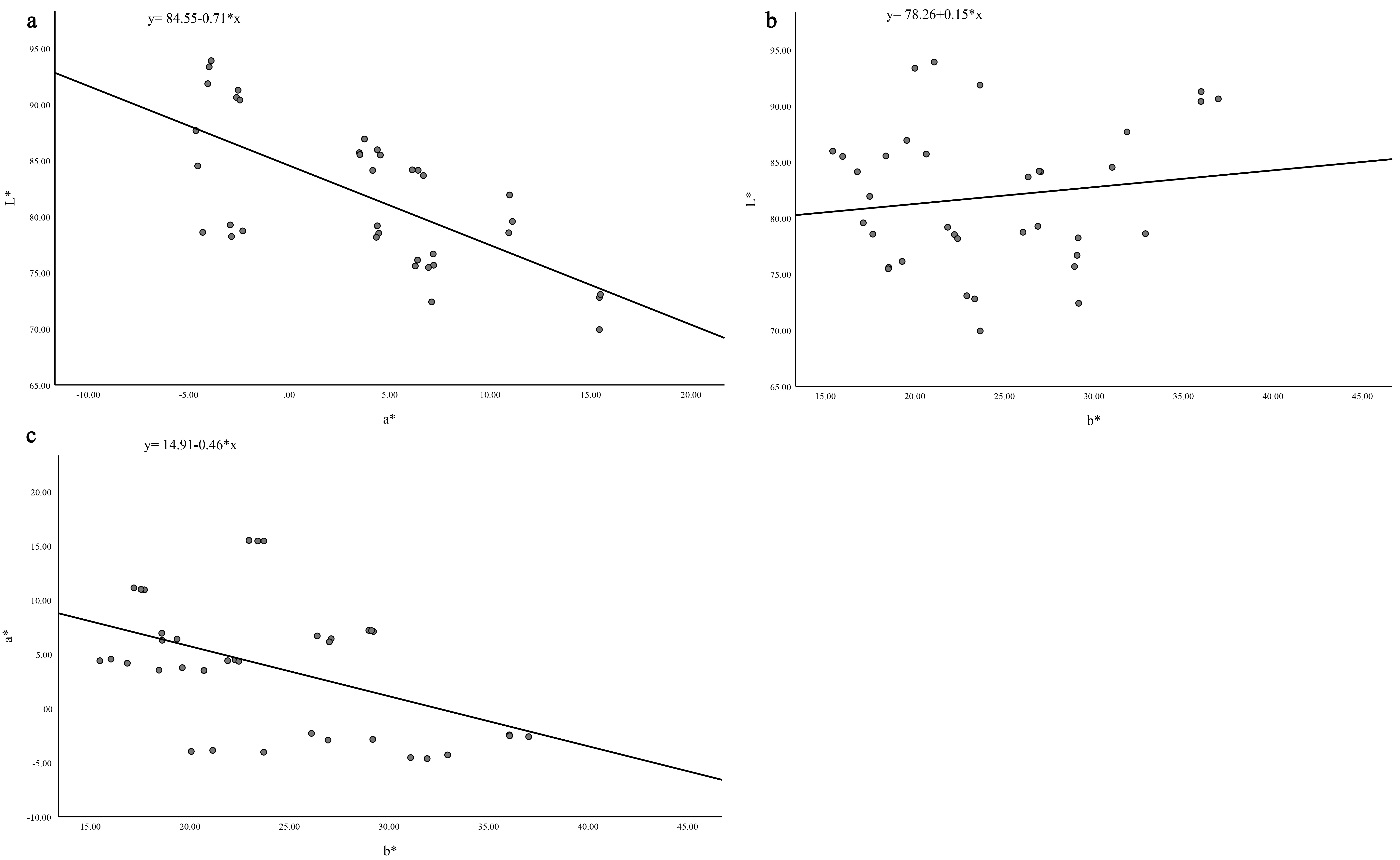

Supplement: Supplementary file 1 [file biology-12-01466-s001.zip › Figure.S1.jpg]

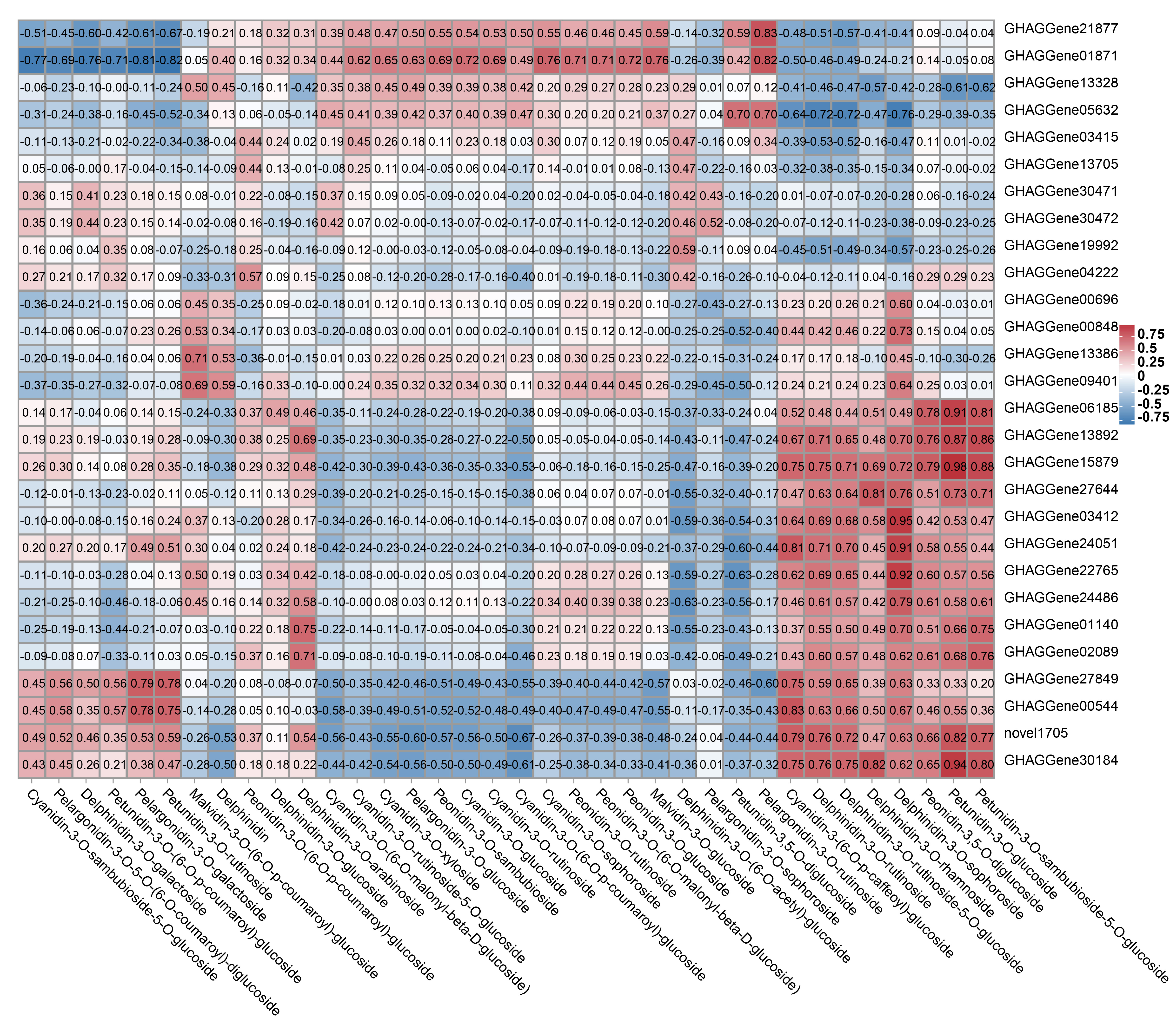

Supplement: Supplementary file 1 [file biology-12-01466-s001.zip › Figure.S2.jpg]

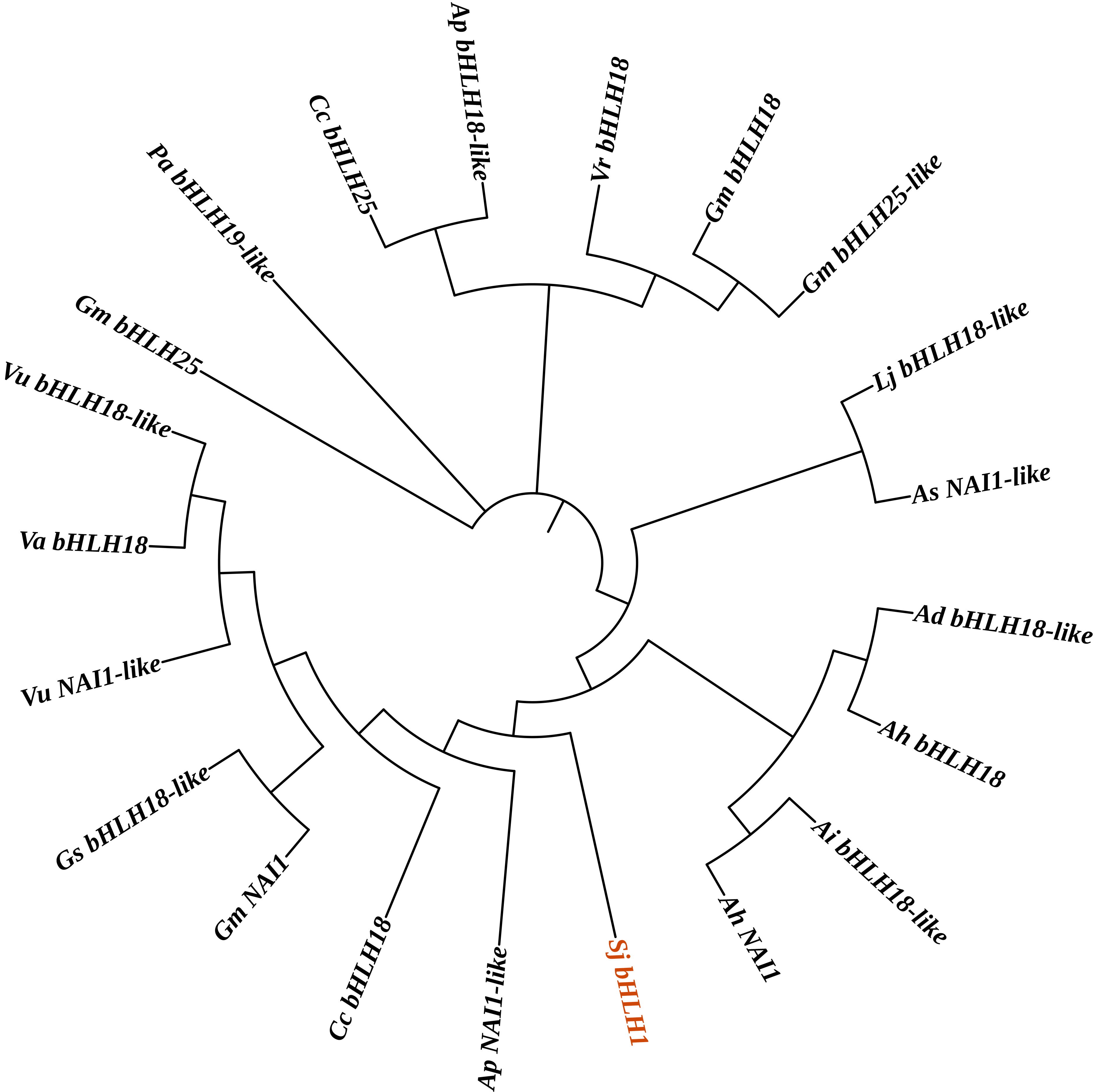

Supplement: Supplementary file 1 [file biology-12-01466-s001.zip › Figure.S3.jpg]
